# Comparison of computer-assisted navigated technology and conventional technology in unicompartmental knee arthroplasty: a meta-analysis

**DOI:** 10.1186/s13018-022-03013-8

**Published:** 2022-02-24

**Authors:** Keteng Xu, Qun Chen, Qing Yan, Qin Wang, Jun Sun

**Affiliations:** 1 Department of Joint Surgery, Huangshan City People’s Hospital, Huangshan, Anhui China; 2 Department of Rheumatology, The First Affiliated Hospital of Soochow University, Suzhou, 215000 Jiangsu China

**Keywords:** Navigation, Knee osteoarthritis, Unicompartmental knee arthroplasty, Meta-analysis

## Abstract

**Background:**

Though unicompartmental knee arthroplasty (UKA) is a useful procedure to treat knee osteoarthritis, it remains a great controversial point as to if navigated systems are able to achieve better accuracy of limb alignment and greater clinic results. Current meta-analysis was conducted to explore if better clinical outcomes and radiographic outcomes could be acquired in the navigated system when compared with conventional procedures.

**Methods:**

We identified studies in the online databases, including Medline, Embase, the Cochrane Library and Web of Science before May 2021. The PRISMA guidelines in this report were strictly followed. Our research was completed via Review Manager 5.4 software.

**Results:**

Fourteen articles were included, involving 852 knees. The present meta-analysis displayed that the navigated system had remarkably improved outcomes in inliers of mechanical axis (MA) (*P* < 0.01), MA in the Kennedy's central zone (Zone C) (*P* = 0.04), inliers of the coronal femoral component (*P* < 0.01), inliers of the coronal tibial component (*P* = 0.005), inliers of the sagittal femoral component (*P* = 0.03), inliers of the sagittal tibial component (*P* = 0.002) and Range Of Motion (ROM) (*P* = 0.04). No significant differences were observed in Oxford Knee Score (OKS) (*P* = 0.15), American Knee Society Knee Score (KSS score) (*P* = 0.61) and postoperative complications (*P* = 0.73) between these 2 groups. Regarding operating time, the navigated group was 10.63 min longer in contrast to the traditional group.

**Conclusion:**

Based on our research, the navigated system provided better radiographic outcomes and no significant difference in the risk of complications with longer surgical time than the conventional techniques. But no significant differences were found in functional outcomes. Because the included studies were small samples and short-term follow-up, high-quality RCTs with large patients and sufficient follow-up are required to identify the long-term effect of the navigated system.

## Background

Osteoarthritis (OA) is the main cause of disability in the elderly across the world, and it affects around 18% of females and 10% of males among people over 60 years old [1–3]. TKA and UKA are common surgical intervention for sufferers with end-stage knee OA [4–6]. Compared with TKA, UKA has less trauma, could keep the normal movement of knee joint, preserves cartilage and bone, has less postoperative complications, recovers quickly and is easier to repair when needed [7, 8]. But at the same time, the risk of malpositioning components is higher because of the limited visualization of anatomical landmarks. The failure of precise component positioning could result in rapid loosening of the component, promoted process of osteoarthritis to the opposite side of the knee and increased revision rate [9–13]. Nowadays, the computer navigated system has been frequently used in UKA procedures [14].

Computer navigated system began to emerge and used in TKA in the late 1990s. Some researches found computer navigated TKA could reduce outliers of alignment and provide good short-term outcomes [4]. Computer navigated system was introduced to UKA surgery by orthopedic surgeons in the early 2000s [14, 15]. This non-image-based system is designed to help orthopedists in improving the accuracy of bony resection and positioning component. By using the infrared camera and the dedicated software, orthopedists can calculate the rotation center of the hip, knee and ankle joints and determine the MA [16, 17]. Previous meta-analyses summarized studies compared the two methods, and reported significantly better radiographic outcomes in patients undergone navigated UKA [18, 19]. But clinical outcomes were not reported. Besides, several new studies were published, including one RCT [7] and two studies [20, 21] with average 9-year follow-up. Due to the need to discuss the clinical outcomes and the increase of high-level literature, it was the purpose of the present report to further assess the differences of radiological and clinical outcomes between the two methods.

## Methods

The study protocol for the present report was accepted by the entire authors prior to the beginning of the literature search, and the protocol was published on the Internet at the PROSPERO (https://www.crd.york.ac.uk/prospero/) with the registration no. CRD42020172412.

### Inclusion and exclusion criteria

The present report follows the inclusion standards below:

(1) Researches contrasted computer navigated UKA and traditional UKA. (2) Clinic or radiography results were not restricted for pooling. (3) Researches written in English were eligible. Researches were ruled out: (1) conferences, reviews, abstracts, case reports, sawbones or cadaveric knee researches. (2) Researches with insufficient data. (3) Redundant publication.

### Literature search

Literature searches were finished to retrieve researches compared between the conventional group and navigational group in the UKA. The search terms were presented below: “navigat*,” “Unicompartmental knee replacement,” “unicompartmental knee arthroplasty,” “unicondylar knee arthroplasty.” Embase, Medline, Web of Science, Cochrane databases were retrieved to search relevant researches before May 2021.

### Data extraction

The data below were separately abstracted from every selected researches by 2 researchers, including publication year, nation, the numbers of sufferers, age, gender, type of component, type of navigation system. The outcomes of our research include clinical outcomes and radiographic outcomes. Radiographic outcomes include inliers of MA (mechanical axis), MA in the Kennedy's zone C, inliers of the coronal femur component, inliers of the sagittal femoral component, inliers of the coronal tibial component and inliers of the sagittal tibial component. The clinical outcomes include OKS scoring, KSS scoring, the WOMAC scoring, ROM, Pain scale (Visual Analogue Scale/Score, VAS), complications (infection, revision, deep venous thrombosis and so on), surgical time (minutes). Whenever there were disagreements, they would be solved via discussing with another researcher (XK).

### Quality evaluation

The non-RCTs research quality was evaluated as per the Downs and Black [22] and the NOS [23] quality evaluation approach. An overall NOS scoring result was 9*, and when the NOS scoring result was > 6*, it was deemed as better quality. A greater scoring was deemed as higher quality. The 12-item scale was employed to evaluate the RCTs quality [24]. Every item was scored “Yes,” “Unclear” or “No.” When a trial with a scoring > 7 “Yes” was deemed as high quality; > 4 but ≤ 7 was deemed as moderate quality, and ≤ 4 was deemed as low quality. Any disagreement was solved by a third researcher.

### Statistical analysis

Statistic data inhomogeneity was assessed via Cochran’s Q statistics. If Q statistics (*P* < 0.10) was deemed as remarkable inhomogeneous among researches, a stochastic effect model was used; otherwise, a fix effect model was employed. If the parametric inhomogeneity was above 85%, the gathering analysis would not be carried out. The outcomes of continuous data were applied to the average deviation with 95% CI. For dichotomous data, the OR was computed via the Mantel–Haenszel approach, average deviation and SMD were considered statistically significant at *P* < 0.05. Data analyses were completed via Review Manager 5.4. Sensitivity analyses were finished to evaluate the outcomes via excluding eligible researches one by one.

## Results

### Study selection

**Fig. 1:**
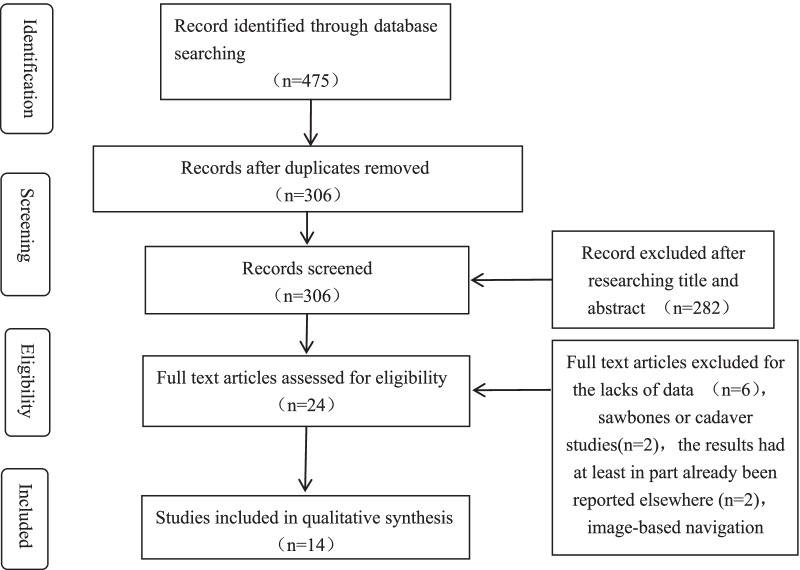
Flowchart of the study selection procedure

By screening the titles and abstracts, 24 studies reaching the inclusion standards were reviewed for full-text screening. Posterior to the full-text eligibility evaluation, certain studies were excluded, as these researches focused on saw bones or cadaveric knees [25, 26] with insufficient data [9, 14, 27–30], and partial data were used in other reports [15, 31]. Eventually, an overall 14 selected reports were described [7, 10, 12, 13, 15, 17, 20, 21, 32–37]. The features of the selected researches in the gathering analysis are presented in Table 1. The navigational group involved 403 knees, and the traditional group involved 449 knees. The screening procedure is presented in Fig. 1.

**Table 1:** Summary of included studies (a)

References	Country	Type	Age, Mean (SD or range)	Gender (F/M)	Keen (n)	Navigated system	Type of prosthesis
Song et al. [21]	Korea	Cohort study	Navigated group: 63.6 (50–79) Conventional group: 64.3 (52–81)	Navigated group: 33/1 Conventional group: 32/2	Navigated group: 34 Conventional group: 34	OrthoPilot	Zimmer
Zhang et al. [7]	China	Randomized controlled trial	Navigated group: 62.4 (5.62) Conventional group: 61.9 (6.11)	Navigated group: 22/18 Conventional group: 22/19	Navigated group: 40 Conventional group: 41	BrainLAB	Zimmer
Manzotti et al. [12]	Italy	Case–control study	Navigated group: 70.9 (7.8) Conventional group: 71.3 (6.8)	Navigated group: 18/13 Conventional group: 18/13	Navigated group: 31 Conventional group: 31	OrthoPilot	DePuy
Valenzuela et al. [37]	USA	Case–control study	Navigated Mean: 65 (48–81) Conventional Mean: 67.3 (49–81)	Navigated group: 28/28 Conventional group: 36/30	Navigated group: 56 Conventional group: 66	Praxim	Zimmer
Weber et al. [17]	Austria	Case–control study	Navigated Mean: 67.9 (44–81.4) Conventional Mean: 69.1 (53.1–79.5)	Navigated group: 11/9 Conventional group: 10/10	Navigated group: 20 Conventional group: 20	OrthoPilot	Univation
Konyves et al. [20]	Australia	Case–control study	Navigated group: 59 (41–78) Conventional group: 61 (44–71)	NR	Navigated group: 15 Conventional group: 15	Stryker	Sulzer
Jung et al. [13]	Korea	Cohort study	Navigated group: 59 (51–76) Conventional group: 56 (46–68)	Navigated group: 13/4 Conventional group: 15/10	Navigated group: 23 Conventional group: 29	Stryker	Oxford
Lim et al. [35]	Australia	Randomized controlled trial	Navigated group: 60 (50–78) Conventional group: 72 (46–85)	Navigated group: 15/15 Conventional group: 11/10	Navigated group: 30 Conventional group: 21	OrthoPilot	FREEDOM
Rosenberger et al. [10]	Austria	Case–control study	Navigated group: 62.9 (6.48) Conventional group: 66.65 (6.48)	Navigated group: 13/7 Conventional group: 9/11	Navigated group: 20 Conventional group: 20	TREON plus	Oxford
Jenny et al. [33]	France	Case–control study	Navigated Mean: 65.6 (48–84) Conventional Mean: 64.5 (44–88)	Navigated group: 34/15 Conventional group: 54/33	Navigated group: 49 Conventional group: 87	OrthoPilot	Aesculap
Keene et al. [34]	Australia	Cohort study	NR	NR	Navigated Mean: 20 Conventional Mean: 20	Ci(DePuy/BrainLAB)	DePuy
Perlick et al. [36]	Denmark	Cohort study	Navigated Mean: 65 (49–73) Conventional Mean: 67 (45–74)	Navigated group: 14/6 Conventional group: 12/8	Navigated group: 20 Conventional group: 20	Ci(DePuy/BrainLAB)	DePuy
Cossey and Spriggins [32]	UK	Cohort study	Navigated group: 55 (41–78) Conventional group: 57 (42–74)	Navigated group: 8/6 Conventional group: 10/4	Navigated group: 15 Conventional group: 15	Stryker	Stryker-Howmedica, Sulzer
Jenny and Boeri [15]	France	Case–control study	NR	NR	Navigated Mean: 30 Conventional Mean: 30	OrthoPilot	Aesculap

NR, no report

### Quality of the included researches

The present report comprises 12 non-RCT [10, 12, 13, 15, 17, 20, 21, 32–34, 36, 37], 2 RCTs [7, 35]. The level of evidence of all eligible studies was at least II level. From the perspective of Downs and Blacks scoring, the entire selected researches were above 15. In NOS, all including non-RCTs had scored ≥ 6*. The non-RCTs report quality is shown in Table 2. The value of weighted kappa for the consensus on those researches between researchers was outstanding (*Κ* = 0.74). Two researches [7, 35] were high quality. The randomization approaches were clearly introduced in the two researches [7, 35]. Random distribution was sufficiently concealed in two researches [7, 35]. No RCTs offered the data of double blinding [7, 35]. None displayed a binding of result evaluation [7, 35]. The RCTs quality is presented in Table 3.

**Table 2:** Summary of included studies (b)

References	Follow-up	Functional evaluation	Mechanical axis	Positioning of the prosthesis	Complications (Navigated group vs. conventional group)	Operating time (SD) [Min]
Song et al. [21]	9 years	HSS, WOMAC	Yes	Yes	1 revision/2 knees were revised	NR
Zhang et al. [7]	2 years	KSS, KSFS	Yes	Yes	No case of complications	Navigated group: 59.4 ± 6.1 Conventional group: 62.1 ± 5.5
Manzotti et al. [12]	6 months	WOMAC, IKS	No	Yes	No case of complications	Navigated group: 47.4 ± 6.1 Conventional group: 35.4 ± 4.4
Valenzuela et al. [37]	NR	NR	Yes	Yes	NR	NR
Weber et al. [17]	18 months	KSS	NR	NR	2 patients were revised to a TKA/1 patient was revised to a TKA	Navigated group: 126.2 ± 21.25 Conventional group: 109.4 ± 18.25
Konyves et al. [20]	Navigated group: 6.9 years Conventional group: 8.9 years	OKS	No	No	NR	NR
Jung et al. [13]	NR	NR	No	Yes	One case of tibial side pin site infection/one case of intraoperative fracture, one case of infection	Navigated group: 98 Conventional group: 82
Lim et al. [35]	1 year	KSS	No	NR	No case of complications	NR
Rosenberger et al. [10]	NR	NR	Yes	NR	No case of complications	Navigated group: 81.8 ± 11.08 Conventional group: 70.85 ± 14.86
Jenny et al. [33]	NR	NR	NR	Yes	No case of complications	NR
Keene et al. [34]	NR	NR	NR	NR	No case of complications	Navigated group: 70 Conventional group: 53
Perlick et al. [36]	NR	NR	Yes	NR	No case of complications	Navigated group: 77 ± 14 Conventional group: 58 ± 11
Cossey and Spriggins [32]	17 months	OKS	Yes	Yes	2 deep venous thrombosis,1 wound infection/1 deep venous thrombosis and 1 superficial wound infection	Navigated group: 81 Conventional group: 58
Jenny and Boeri [15]	3 months	NR	Yes	Yes	NR	Navigated group: 86 Conventional group: 67

KSS, Knee Society Score; WOMAC, Western Ontario and McMaster Osteoarthritis Index; IKS, International Knee Society; OKS, Oxford Knee Score

Yes, Better positioning of the prosthesis/mechanical axis through navigation; No, no difference; NR, no report

**Table 3:** Description of the quality of included non-RCTs studies

References	Country	Type	Level of evidence	Study quality	NOS Scale				
				Downs and Black Score	Selection	Comparability	Expose	Outcome	Total score
Song et al. [21]	Korea	Cohort study	III	16	***	**	–	**	*******
Manzotti et al. [12]	Italy	Case–control study	II	21	***	**	**	–	*******
Valenzuela et al. [37]	USA	Case–control study	II	17	***	**	*	–	******
Weber et al. [17]	Austria	Case–control study	II	23	**	*	**	–	******
Konyves et al. [20]	Australia	Case–control study	II	19	***	**	**	–	*******
Jung et al. [13]	Korea	Cohort study	III	20	***	**	–	**	*******
Rosenberger et al. [10]	Austria	Case–control study	II	16	***	**	**	–	*******
Jenny et al. [33]	South-Korea	Case–control study	II	16	**	**	**	–	******
Keene et al. [34]	France	Cohort study	III	17	***	*	–	**	******
Perlick et al. [36]	Australia	Cohort study	II	18	***	**	–	*	******
Cossey and Spriggins [32]	Denmark	Cohort study	II	19	***	*	–	**	******
Jenny and Boeri [15]	UK	Case–control study	III	18	***	**	**	–	*******

### Inliers of MA

**Fig. 2:**
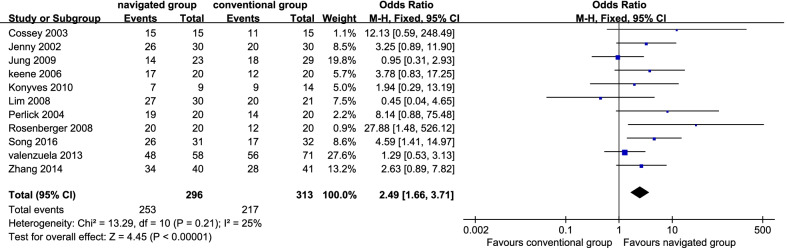
Forest plot diagram showed the mean difference in Oxford Knee Score (OKS score) between navigated group and conventional group

Eleven studies [7, 10, 13, 15, 20, 21, 32, 34–37] reported the rate of inliers of MA. There were 253 patients (85.47%) in the navigated group and 217 sufferers (69.33%) in the traditional group. No significant inhomogeneity was identified (*P* = 0.21; *I*^2^ = 25%); hence, the fix effect model was employed. It displayed that the navigated group was remarkably more satisfactory than the conventional group in the rate of inliers of MA (OR 2.49, 95% CI 1.66–3.71, *P* < 0.01; Fig. 2).

### MA in the Kennedy's Zone C

**Fig. 3:**
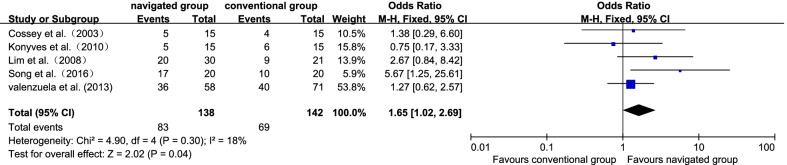
Forest plot diagram showed the mean difference in American Knee Society Knee Score (KSS score) between navigated group and conventional group

Five studies [20, 21, 32, 35, 37] reported MA in the Kennedy's Zone C. There were 83 sufferers (60.14%) in the navigated group and 69 sufferers (48.59%) in the traditional group. No significant inhomogeneity was identified (*P* = 0.30; *I*^2^ = 18%); thereby, the fix effect model was employed. It displayed that the navigated group had a remarkably more satisfactory result in the rate of MA in the Kennedy's Zone C (OR 1.65, 95% CI 1.02–2.69, *P* = 0.04; Fig. 3).

### Inliers of the coronal femoral component

**Fig. 4:**
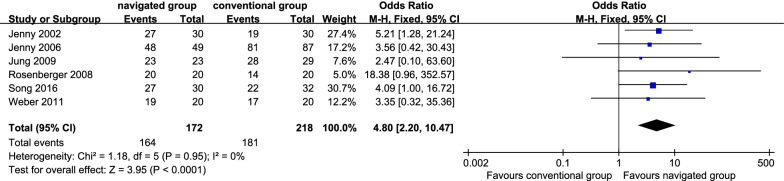
Forest plot diagram showed the mean difference in Range Of Motion (ROM) between navigated group and conventional group

Six studies [10, 13, 15, 17, 21, 33] reported inliers of the coronal femoral component. There were 164 sufferers (95.38%) in the navigated group and 181 sufferers (83.03%) in the traditional group. There was no significant inhomogeneity among the researches (*P* = 0.95; *I*^2^ = 0%), so the fix effect model was employed. It displayed that the navigated group was remarkably more satisfactory in the rate of inliers of the coronal femoral component than the conventional group (OR 4.80, 95% CI 2.20 to 10.47, *P* < 0.01; Fig. 4).

### Inliers of the sagittal femoral component

**Fig. 5:**
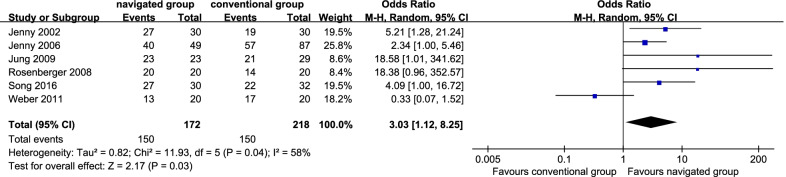
Forest plot diagram showed the complications between navigated group and conventional group

Six studies [10, 13, 15, 17, 21, 33] reported inliers of the sagittal femoral component. There were 150 patients (87.21%) in the navigated group and 150 sufferers (68.81%) in the traditional group. Heterogeneity was remarkable in the studies (*P* = 0.04.1; *I*^2^ = 58%); thereby, the random effect model was employed. It displayed that the navigated group was remarkably more satisfactory in the rate of the sagittal femoral component than the traditional one (OR 3.03, 95% CI 1.12 to 8.25, *P* = 0.03; Fig. 5).

### Inliers of the coronal tibial component

**Fig. 6:**
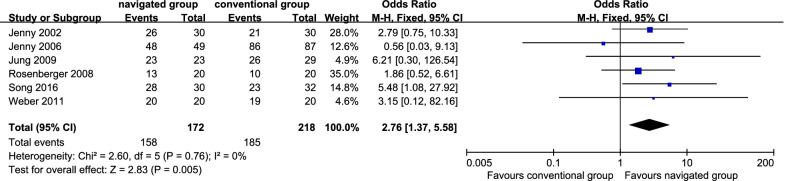
Forest plot diagram showed the mean difference in Pain scale (Visual Analogue Scale/Score, VAS) between navigated group and conventional group

Six studies [10, 13, 15, 17, 21, 33] reported inliers of the coronal tibial component. There were 158 patients (91.86%) in the navigated group and 185 sufferers (84.86%) in the traditional group. No significant inhomogeneity was identified (*P* = 0.76; *I*^2^ = 0%); thereby, the fix effect model was employed. It displayed that the navigated group was remarkably more satisfactory in the rate of inliers of the coronal tibial component than the traditional one (OR 2.76, 95% CI 1.37 to 5.58, *P* = 0.005; Fig. 6).

### Inliers of the sagittal tibial component

**Fig. 7:**
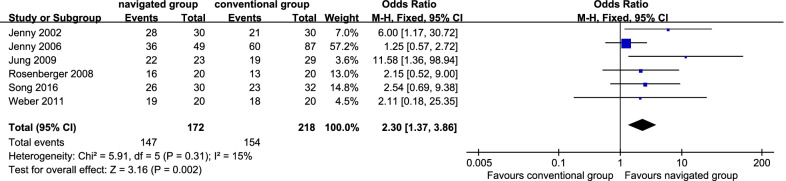
Forest plot diagram showed the proportion of tibiofemoral mechanical axis of satisfactory ranges between navigated group and conventional group

Six studies [10, 13, 15, 17, 21, 33] reported inliers of the sagittal tibial component. There were 147 patients (85.47%) in the navigated group and 154 sufferers (70.64%) in the traditional group. No remarkable inhomogeneity was identified (*P* = 0.31; *I*^2^ = 15%); thereby, the fix effect model was employed. It displayed that the navigated group was remarkably more satisfactory in the rate of inliers of the sagittal tibial component than the traditional one (OR 2.30, 95% CI 1.37 to 3.86, *P* = 0.002; Fig. 7).

### OKS score

**Fig. 8:**
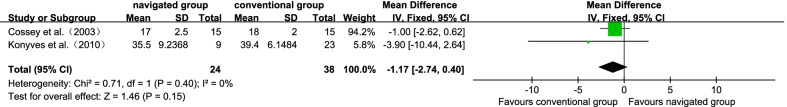
Forest plot diagram showed the proportion of coronal femoral component of satisfactory ranges between navigated group and conventional group

Two studies [20, 32] contrasted the OKS score. These data were gathered for analyses. No remarkable inhomogeneity existed among the researches (P = 0.40; *I*^2^ = 0); hence, the fix effect model was employed. It displayed no remarkable diversity in the OKS score between these 2 groups (MD = − 1.17, 95% CI − 2.74 to 0.40, *P* = 0.15; Fig. 8).

### KSS score

**Fig. 9:**
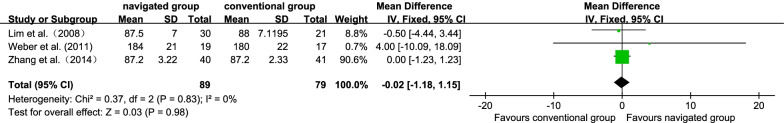
Forest plot diagram showed the proportion of sagittal femoral component of satisfactory ranges between navigated group and conventional group

Three studies [7, 17, 35] compared the KSS score. These data were gathered for analyses. No remarkable inhomogeneity existed among the researches (*P* = 0.83; *I*^2^ = 0); hence, the fix effect model was employed. There was not remarkable diversity in the KSS score between these 2 groups (MD = − 0.02, 95% CI − 1.18 to 1.15, *P* = 0.61; Fig. 9).

### WOMAC score

In the articles we included two reported results of the WOMAC score [12, 21]. The inhomogeneity was above 85% (*I*^2^ = 95%); for that reason, the overall effects of this parameter were not implemented (Table 4).

**Table 4:** RCTs studies quality

RCTs	Randomized adequately^a^	Allocation concealed	Patient blinded	Care provider blinded	Outcome assessor blinded	Acceptable drop-out rate^b^	ITT Analysis^c^	Avoided selective reporting	Similar baseline	Similar or avoided cofactor	Patient compliance	Similar timing	Quality^d^
Zhang et al. [7]	Yes	Yes	Yes	Unclear	No	Yes	No	Yes	Yes	Unclear	Yes	Unclear	High
Lim et al. [35]	Yes	Yes	Yes	Unclear	No	Yes	No	Yes	Yes	Unclear	Yes	Yes	High

^a^Only if the method of sequence made was explicitly introduced could get a “Yes”

^b^Drop-out rate < 20% could get a “Yes,” otherwise “No”

^c^ITT = intention-to-treat, only if all randomized participants were analyzed in the group, they were allocated to could receive a “Yes”

^d^“Yes” items more than 7 means “High”; more than 4 but no more than 7 means “Moderate”; no more than 4 means “Low”

### ROM

**Fig. 10:**
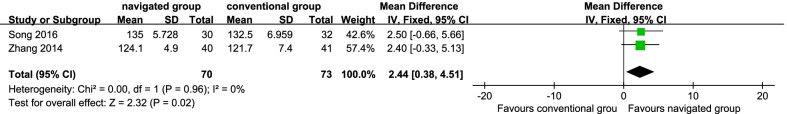
Forest plot diagram showed the proportion of coronal tibial component of satisfactory ranges between navigated group and conventional group

Two studies compared [7, 21] the ROM. Thereby, our team accepted them for the gathering analysis. No significant inhomogeneity was identified between the researches (*P* = 0.96; *I*^2^ = 0%); hence, the fix effect model was employed. The gathered outcomes displayed that the navigational group exhibited a remarkable increase in ROM (MD = 2.44, 95% CI 0.38 to 4.51, *P* = 0.04; Fig. 10).

### VAS

Two researches [7, 12] involved the VAS. The inhomogeneity was above 85% (*I*^2^ = 87%); for that reason, the overall effects of this parameter were not implemented.

### Complications

**Fig. 11:**
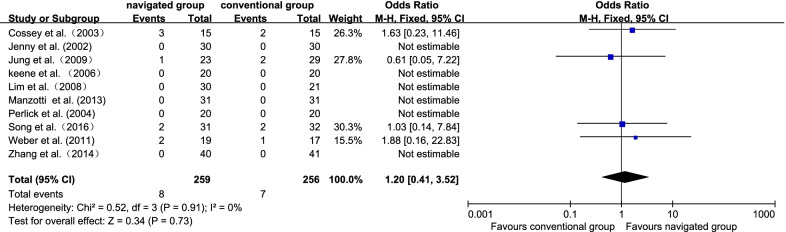
Forest plot diagram showed the proportion of coronal tibial component of satisfactory ranges between navigated group and conventional group

Ten studies [7, 12, 13, 15, 17, 21, 32, 34–36] displayed complicating diseases. The navigated group had 8 sufferers (2.76%), and the traditional group had 7 sufferers (2.42%). No significant inhomogeneity was identified (*P* = 0.91; *I*^2^ = 0%); hence, the fix effect model was employed. It displayed no significant diversity in the rate of complications between these 2 groups (OR 1.20, 95% CI 0.41 to 3.52, *P* = 0.73; Fig. 11).

### Surgical time (minutes)

Five articles [7, 10, 12, 17, 36] reported the outcomes of surgical time. The navigated group was 10.63 min longer in contrast to the traditional one. The inhomogeneity was above 85% (*I*^2^ = 95%); for that reason, the overall effects of this parameter were not implemented.

### Sensitivity analyses

One research was individual removed every time to identify the influence on the gathered MD or OR. The outcomes displayed that no research was able to remarkably influence the gathered MD or OR in this gathering analysis.

## Discussion

The discovery of our gathering analysis was that using the navigation system significantly improved the precision of rebuilding the MA and placing components in UKA. Regarding clinical outcomes, ROM of navigation group was significantly better than that of conventional group. Besides, the operative duration of the navigated group was 10.63 min longer in contrast to the traditional one. But no remarkable diversity was identified in the OKS scoring, the KSS scoring and complications.

It is generally accepted that the accuracy of radiographic outcomes is crucial for knee pain relief, implant survival and long-term functional outcomes [11, 18, 29, 38, 39]. Our meta-analysis evaluated radiographic outcomes by the inliers of MA, the position of femoral components, the position of tibial components and the rate of MA in the Zone C. Unlike previous studies [18, 19], our study added the rate of MA in the Zone C, which is recognized as an acceptable range for optimizing long-term results, to further evaluate the accuracy of radiographic outcomes between the two methods [40]. The present gathering analysis discovered that the navigated system remarkably reduced the risk of MA outliers and components, improved the rate of MA falling in Zone C. Song et al. also found the use of the navigated system contributed to significantly improved rate of desired MA and components placement over an average 9-year follow-up [21]. Although some researchers have found no significant difference in the radiographic outcomes, they have identified possible reasons for the conflicting outcomes, such as small samples, short follow-up, inaccurate navigated system and software incompatibility [12, 35]. However, these studies also favored using the navigated system when inexperienced orthopedists performed UKA.

There is much debate about the ideal position of the tibial prosthesis. Some orthopedists are happy to maintain neutral correction of tibial axis, some insist on slight under correction, while certain orthopedists are favor to reconstruct the initial anatomy structure without worrying about the tibial axis [11, 14]. In the included studies, the definition of the ideal tibial slope is also different. It was 3-degree in Song’s study [21], 5-degree in Rosenberger’s study [10] and 7-degree posterior to the 2-degree anterior in Weber’s study [17], whereas Perlick et al. favored tibial slope adapted according to individual patient’s situation [36]. Researches are required to discuss the ideal position of the tibial prosthesis. Determining the ideal position of the tibial prosthesis can more accurately compare the advantages and disadvantages of the two groups.

Functional outcomes are important indicators to evaluate the effect of UKA. Our studies evaluated functional outcomes based on the OKS score, KSS score and ROM. Although the navigated group had significantly better outcome in ROM, no remarkable diversity existed in OKS score and KSS score between the two groups. The introduction of the navigated system bases on the assumption that the optimal MA and components location are closely associated with satisfying functional outcomes. However, our results contradicted the assumption. The small sample of some included studies may contribute to it. Only two studies [20, 32] reported the OKS and three studies [7, 17, 35] reported the KSS. Besides, the effect of fine MA on the postoperative function needs a long period to be obvious [7, 17, 20, 32, 35]. Song et al. had reported the navigated group had more accuracy radiographic outcomes, significantly better results in functional scale-Hospital for Special Surgery (HSS) and pain scale scores were found over an average 9-year follow-up. Saragaglia et al. also supported the navigated system had better outcomes in a long-term follow-up [14].

In our meta-analysis, no remarkable diversity existed in the rate of postoperative complicating diseases (infection, revision, deep venous thrombosis and so on) between these 2 groups. Chona et al. contrasted the ratio of complications between the two methods using the Medicare database (included 9,228 UKAs) [16], and they also found no significant difference at 30 days, 90 days and 2 years. Therefore, we speculated there may be no remarkable diversity between these 2 methods in the prevalence of complications during short-term follow-up. But it is hard to tell the difference between long-term follow-up, because most of the studies included were short-term follow-up. A mean follow-up time of 10 years may be sufficient to determine whether there is a diversity in the incidence of complicating diseases between these 2 groups [41].

Some studies have reported on the disadvantages of the navigation system, including the longer operating time [7, 10, 12, 17, 36]. Certain extra steps to fasten the tracking device to the limb and register anatomical signs may be the cause of longer surgical time [12, 21]. But the navigated system has the potential advantage of improving the precision of implant positioning. Thus, the additional time may be worth it [18].

The cost-effectiveness between the two methods has not been studied. Another drawback of the navigated system is that the cost may be higher than the conventional method. Novak et al. speculated the expenditure of the navigated system in TKA to be $1500 for every patient [16]. We considered the following items ought to consider the aspect of expenditure, such as revisions, potential complications and functional outcomes.

The critical aspects for studies in the future:

(1) Further studies are needed to assess the long-term clinic outcomes of UKA. (2) The CT scanning might be more satisfactory because it offers a more precise assessment of the cement polyethylene. (3) Prospective studies are required to compare the hospital and socioeconomic cost-effectiveness between the two methods. (4) Further researches are necessary to determine the ideal target value of the tibial component. (5) More approaches, like the robotic systems, ought to be explored during the comparison between these 2 approaches.

Certain flaws ought to be taken into consideration. (1) The proofs of certain selected researches were not sufficient and merely two RCTs were selected herein, which might affect the accurateness of the gathered outcomes. (2) Great inhomogeneity of certain outcomes was discovered in the present gathering analysis. The diverse navigation systems, operative technologies, calibration of targets, component types, medial or lateral UKA, fixed or mobile bearing, cemented or non-cemented component and radiography measurement approaches were thought to be the causes. Nevertheless, our team failed to carry out sub-group assay of those elements owing to insufficient data. (3) Little research had adequate postoperation follow-up time, which was the primary cause hindering us from acquiring more stringent and persuasive results. (4) The specimen size of some selected researches was comparatively small, particularly the study of Konyves et al. (5) There are publication biases.

## Conclusion

Based on our research, the navigated system provided better radiographic outcomes and no significant difference in the risk of complications with longer surgical time than the conventional techniques. But no significant differences were found in functional outcomes. Because the included studies were small samples and short-term follow-up, high-quality RCTs with large patients and sufficient follow-up are required to identify the long-term effect of the navigated system.

## Data Availability

All data generated or analyzed during this study are included in this published article.

## Abbreviations

UKA: Unicompartmental knee arthroplasty
TKA: Total knee arthroplasty
OKS: Oxford Knee Score
KSS score: American Knee Society Knee Score
WOMAC score: The western Ontario and McMaster Universities Osteoarthritis Index
MA: Mechanical axis
ROM: Range Of Motion
HSS: The Hospital for Special Surgery
NOS: Newcastle–Ottawa Scale
RCTs: Randomized controlled trials
CI: Confidence interval
OR: Odd ratio
MD: Mean difference
